# Supplementary material for: SMRT sequencing of the full-length transcriptome of the white-backed planthopper Sogatella furcifera
Source: PeerJ. 2020 Jun 9;8:e9320. doi: 10.7717/peerj.9320 (PMC7292024; doi:10.7717/peerj.9320)
Supplement: Supplemental Information 1 — i) sample: sample name. ii) Total reads: the number of reads after data filtering. iii) Total mapped: the number of identical sequences that could be compared to the genome. iv) Unmapped:the number of sequences that could not be compared to the genome. v) Multiple mapped: the number of identical sequences with multiple alignment positions on the reference genome. vi) Uniquely mapped: the number of identical sequences with one positions on the reference genome. vii) Reads map to ’+’: the number of identical sequences alignment to positive strand of reference genome. viii) the number of identical sequences alignment to negative strand of reference genome. [file peerj-08-9320-s001.docx]

| **Sample name** | **Total reads** | **Total mapped** | **Unmapped** | **Multiple mapped** | **Uniquely mapped** | **Reads map to '+'** | **Reads map to '-'** |
| --- | --- | --- | --- | --- | --- | --- | --- |
| *S. furcifera* | 251,109 | 162,699 (64.79 %) | 88,410 (35.21 %) | 31,535  (12.56 %) | 131,164  (52.23 %) | 85,931  (34.22 %) | 45,233  (18.01 %) |
